# Clinical Characteristics Are Similar across Type A and B Influenza Virus Infections

**DOI:** 10.1371/journal.pone.0136186

**Published:** 2015-09-01

**Authors:** Anne Mosnier, Saverio Caini, Isabelle Daviaud, Elodie Nauleau, Tan Tai Bui, Emmanuel Debost, Bernard Bedouret, Gérard Agius, Sylvie van der Werf, Bruno Lina, Jean Marie Cohen, GROG network

**Affiliations:** 1 Open Rome (Organize and Promote Epidemiological Network), Paris, France; 2 General Practitioner or Paediatrician, contributor of the GROG network, Paris, France; 3 Laboratoire de Virologie, Centre Hospitalier Universitaire de Poitiers, Poitiers, France; 4 Centre National de Référence des virus influenza, Génétique moléculaire des virus respiratoires, Institut Pasteur, CNRS UMR 3569, Université Paris Diderot Sorbonne Paris-Cité, Paris, France; 5 Centre National de Référence des virus influenza, CBPE, Hospices Civils de Lyon et Virpath, Université Claude Bernard Lyon, Lyon, France; Public Health Agency of Canada, CANADA

## Abstract

**Background:**

Studies that aimed at comparing the clinical presentation of influenza patients across virus types and subtypes/lineages found divergent results, but this was never investigated using data collected over several years in a countrywide, primary care practitioners-based influenza surveillance system.

**Methods:**

The IBVD (Influenza B in Vircases Database) study collected information on signs and symptoms at disease onset from laboratory-confirmed influenza patients of any age who consulted a sentinel practitioner in France. We compared the clinical presentation of influenza patients across age groups (0–4, 5–14, 15–64 and 65+ years), virus types (A, B) and subtypes/lineages (A(H3N2), pandemic A(H1N1), B Victoria, B Yamagata).

**Results:**

Overall, 14,423 influenza cases (23.9% of which were influenza B) were included between 2003–2004 and 2012–2013. Influenza A and B accounted for over 50% of total influenza cases during eight and two seasons, respectively. There were minor differences in the distribution of signs and symptoms across influenza virus types and subtypes/lineages. Compared to patients aged 0–4 years, those aged 5–14 years were more likely to have been infected with type B viruses (OR 2.15, 95% CI 1.87–2.47) while those aged 15–64 years were less likely (OR 0.83, 95% CI 0.73–0.96). Males and influenza patients diagnosed during the epidemic period were less likely to be infected with type B viruses.

**Conclusions:**

Despite differences in age distribution, the clinical illness produced by the different influenza virus types and subtypes is indistinguishable among patients that consult a general practitioner for acute respiratory infections.

## Introduction

Influenza is clinically characterized by nonspecific signs (detected by the physician during clinical examination) and symptoms (defined as sometimes experienced and reported by the patient) that are common to other infections, such as sudden onset, fever, malaise, headache and cough [1]. All age groups are affected, with highest incidence among those younger than 25 years [2]. Influenza is usually a self-limiting disease and most affected people recover in 3–5 days, although cough and malaise may persist for up to two weeks [3]; it can be complicated and life-threatening among patients who are vulnerable due to coexisting chronic diseases, weakened immune systems, or other underlying conditions [4].

Several studies have investigated whether the clinical presentation of influenza differs depending on the type of virus that caused the illness, most of them [5–12] focusing on paediatric populations and specific influenza seasons. No difference was observed in most cases [13]; however, some distinct features were consistently reported, like for influenza B a higher incidence of myalgia among children that are old enough to report subjective symptoms.

Far less reports are available on clinical manifestations by influenza type and subtype among adults. Medically-attended influenza A(H3N2) patients were found to be more severely ill than patients infected with other virus (sub)-types [14–15]; influenza B patients presented more frequently with gastrointestinal symptoms [14]; and few or no differences were observed in the clinical presentation of out- and in-patients with pandemic vs. seasonal A(H1N1) influenza [16–17]. However, no consistent differences were observed in the clinical presentation of outpatient-attended influenza patients by virus type [18–19], except for fewer signs and symptoms among patients with pandemic A(H1N1) vs. A(H3N2) or type B virus infection. To the best of our knowledge, no studies were carried out that explicitly aimed at comparing the clinical presentation of influenza cases by virus type and subtype using data collected over several consecutive years in the context of a countrywide, primary care practitioners (general practitioners [GPs] and paediatricians)-based influenza sentinel surveillance system.

The IBVD (Influenza B in Vircases Database) study was conducted with the purpose of feeding knowledge on influenza B disease in terms of demographics, clinical presentation, and treatments administered. The project, led by Open Rome, a private research organization, was based on routine influenza surveillance data that are being collected since season 2003–2004 by the GROG (Regional Group for Influenza Surveillance) national network in France from outpatients who consulted a primary care practitioner due to an acute respiratory illness (ARI), and subsequently had a diagnosis of lab-confirmed influenza. We present here a comparison of the clinical presentations of influenza cases by virus type and subtype/lineage for the period 2003–2004 to 2012–2013.

## Methods

### The GROG Network

Established in 1984 [20], the GROG is a network of sentinel general practitioners and paediatricians (referred to as “sentinel practitioners” below) that collects clinical and virological information on influenza activity in outpatients in France from October through April. At the national level, the epidemic period used for the purpose of surveillance is defined as the period during which at least two clinical indicators (including ARI by encounters) are increased by at least 20% compared to the October baseline, AND several similar influenza viruses (same type and subtype) have been detected or isolated in different regions (the % of positive isolates being usually above 10%).

The number of participating sentinel practitioners was around 500 (80% were GPs) during the ten seasons, covering 21 of the 22 regions of metropolitan France.

### Selection of Patients and Data Collection in the GROG Network

Each sentinel practitioner collects information and provides nasal/pharyngeal swabs from a subset of their patients presenting with ARI within 48 hours of onset of symptoms and suspected for having influenza. The definition of ARI is as follows: sudden onset of at least one respiratory sign or symptom (cough, sore throat, shortness of breath, coryza…) AND at least one systemic sign or symptom suggestive of an acute infectious disease (fever, fatigue, headache, malaise…).

The data collected in the standardized questionnaire (see S1 Fig) are demographics; signs and symptoms at medical encounter; influenza vaccination status; presence of underlying conditions with indications for influenza vaccination; antibiotics prescription; antivirals prescribed or taken during the two weeks prior to consultation; number of consultations with the practitioner and hospitalizations during the previous 12 months; and whether the patient was referred to a hospital following the consultation with the sentinel practitioner.

Collected data of ARI cases that are swabbed and eventually diagnosed with influenza type A or B viruses are entered into a database named “Vircases”.

### Study Database and exclusion criteria

Some exclusion criteria were previously applied to the GROG Database to construct the IBVD Database. We excluded from the analyses vaccinated cases and patients whose age was missing. To limit the influence of others factors on clinical presentation, we also excluded patients co-infected with two different influenza viruses or with an influenza virus and another respiratory virus, as well as those with antivirals taken up to 15 days prior to consultation.

Due to the emergence of the new A(H1N1)pdm09 influenza virus in 2009 and in order not to burden the Results section too much, we chose not to include the seasonal (pre-pandemic) A(H1N1) influenza virus subtype in the clinical presentation analysis as it is no longer circulating since the 2009 pandemic.

When there were fewer than 40 cases due to one influenza type or subtype during a given season, such cases were also not included in the IBVD database, as the focus was on virus strains that were dominant in the season.

### Sampling Procedures and Laboratory Diagnosis

Specimens were transported to the laboratory by post (along with a copy of the questionnaire used for data collection), with a triple packaging system following the international guidelines for the transport of infectious substances (category B, classification UN 3373). The virological analyses were performed in one of the virology laboratories collaborating with the GROG network, which is in most cases the French National Influenza Centre (NIC) (Institut Pasteur, Paris, and Hospices Civils, Lyon).

The specimens of swabbed ARI patient were analyzed for the presence of influenza viruses and if positive, in most cases, the virus type and subtype (for influenza A viruses) were determined. Following the WHO collaborating centres recommendations for virus isolation, subtyping and lineage characterization, the influenza B virus lineage was characterized only on a random subset of specimens that were sent to the NIC [21]. Until 2008–2009, laboratory confirmation essentially relied on enzyme immunoassays for determination of the virus type, and on isolation in cell culture followed by hemagglutination inhibition assays using specific polyclonal sera for determination of the virus type, sub-type and for antigenic characterization. Since 2009–2010, the laboratories have been mainly performing real-time reverse transcriptase polymerase chain reaction (RT-PCR) tests for virus detection, (sub)-typing and determination of influenza B lineage [22]. According to the terms of reference of the NICs and WHO collaborating centres, laboratory procedures for strain typing were validated at the WHO Collaborating Centre for Reference and Research on Influenza of the National Institute for Medical Research at Hill Mill, UK, using a ten percent sample.

### Statistical Analysis

We analysed the distribution of signs and symptoms across influenza virus types, subtypes and lineages comparing: B vs. A, B vs. A(H3N2), B vs. A(H1N1)2009, and B Victoria vs. B Yamagata. As age is a strong determinant of clinical presentation, each comparison was performed separately for each of the age groups used in European influenza surveillance: 0–4 years, 5–14 years, 15–64 years, 65+ years. Myalgia and cephalalgia were not considered among patients aged 0–4 years as children of this age cannot report their subjective symptoms. For comparison of categorical and continuous variables, the chi-square test and the Wilcoxon rank sum test were used, respectively. We used the Kolmogorov-Smirnov test, the t-test and the Wilcoxon rank sum test to compare the distribution, the mean and, respectively, the median age of influenza patients infected with different influenza viruses.

A multivariable logistic regression model was fitted to look for individual, clinical or epidemiological characteristics that would help discriminate between influenza B and A patients. Variables included were gender, age group, number of signs and symptoms (<7/≥7), season, and week of diagnosis (during or outside the epidemic period).

All analyses were performed by using Microsoft Excel and STATA version 11 (STATA Corp., TX, USA). All statistical tests were two-sided, and considered as significant for p<0.05.

### Ethical Aspects and Consent

In accordance with French ethical rules, verbal informed consents are sufficient for patients participating in an epidemiological surveillance study. Practitioner’s participation in the study is advertised in the participants waiting room. Each practitioner takes responsibility for collecting the oral consent from ARI cases, at the moment of swab taking after providing appropriate information on the general surveillance characteristics. The consent is given orally but is registered in the data collection form accompanying the swab. In accordance with applicable laws and regulations, no clearance of an Ethics Committee is required in France for the retrospective analysis of anonymised data collected within routine influenza surveillance schemes.

A limited access to the GROG database is granted to Open Rome through a dedicated research contract written for this study. The ownership of the data remains with the GROG.

## Results

**Fig 1:**
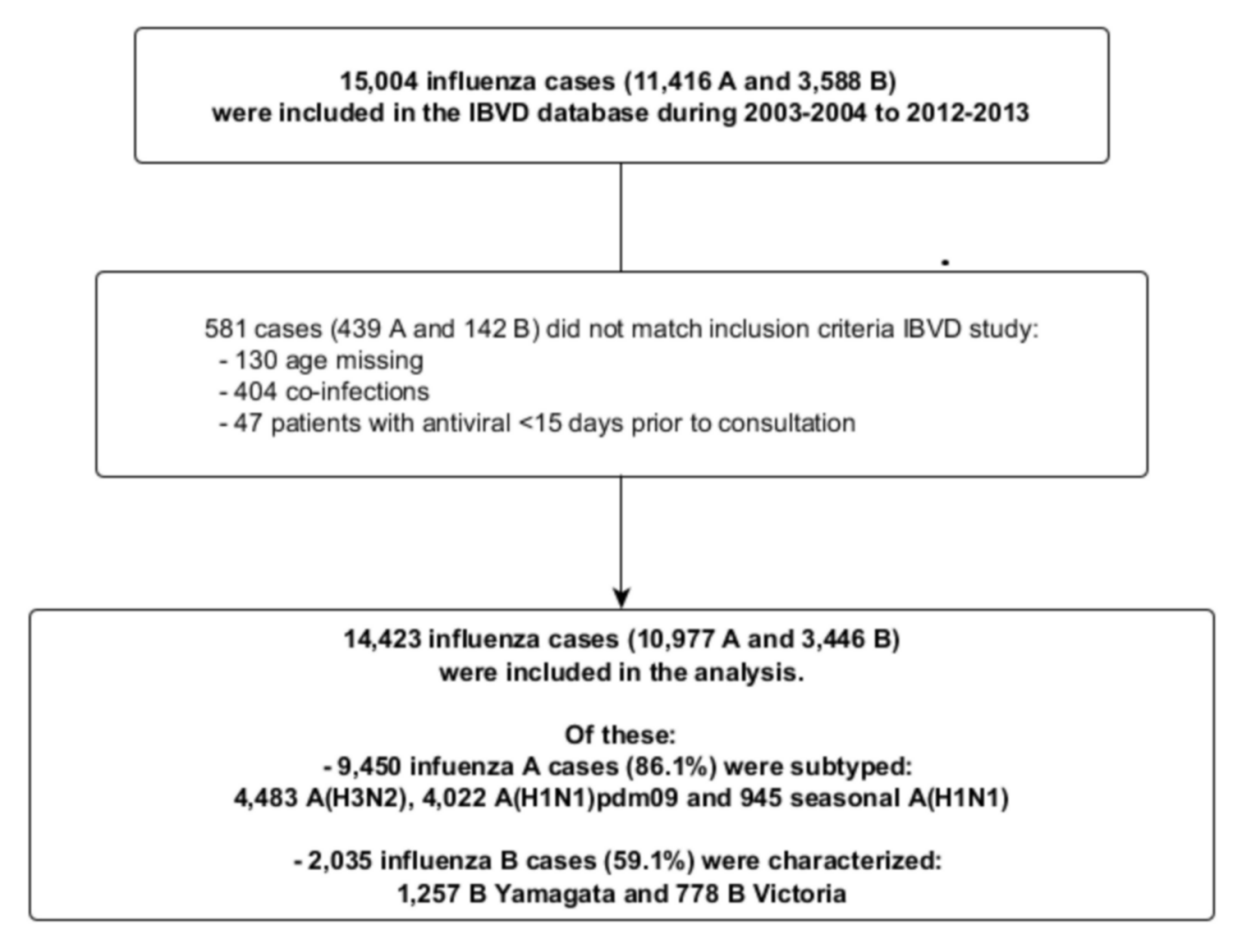
Flow chart of the influenza A and B patients included in the IBVD (Influenza B in Vircases Database) study.

Overall, 15,004 influenza cases (11,416 A and 3,588 B) were included in the IBVD database during the study period: of these, 581 cases (439 A and 142 B) were dropped from the analysis according to exclusion criteria (Fig 1).

Finally 14,423 influenza cases were included in the study (Table 1). 10,977 A (of which 9,450 subtyped, 86.1%) and 3,446 B (of which 2,035 characterized, 59.1%) influenza cases were kept for the analysis, including 4,483 A(H3N2), 4,022 A(H1N1)pdm09, 945 seasonal A(H1N1), 778 B Victoria and 1,257 B Yamagata.

**Table 1:** Number of swabbed ARI and influenza cases, and percentages attributable to different influenza virus types and subtypes/lineages, in the IBVD (Influenza B in Vircases database) study. France, 2003–2004 to 2012–2013.

Season	Duration (week start-end of influenza epidemic)	ARI cases	Influenza cases	A^(a)^	B^(a)^	A(H1N1)	A(H3N2)	A(H1N1) pdm09	A not subtyped	B Victoria	B Yamagata	B not characterized
**2003–2004**	7 (47/2003-01/2004)	2432	874	100.0%	0.0%	0.7%	88.8%	0.0%	10.5%	-	-	-
**2004–2005**	10 (03/2005-12/2005)	3262	975	90.3%	9.7%	9.5%	61.5%	0.0%	29.0%	5.3%	51.6%	43.2%
**2005–2006**	9 (03/2006-11/2006)	4139	840	37.6%	62.4%	79.4%	0.0%	0.0%	20.6%	62.2%	7.6%	30.2%
**2006–2007**	5 (04/2007-08/2007)	3841	945	100.0%	0.0%	2.0%	72.7%	0.0%	25.3%	-	-	-
**2007–2008**	7 (02/2008-08/2008)	4254	1240	65.2%	34.8%	68.9%	0.0%	0.0%	31.1%	0.7%	58.3%	41.0%
**2008–2009**	10 (50/2008-07/2009)	3929	1365	84.8%	15.2%	2.4%	70.6%	0.0%	26.9%	49.8%	5.8%	44.4%
**2009–2010**	7 (47/2009-53/2009)	7630	2749	100.0%	0.0%	0.0%	0.0%	95.9%	4.1%	-	-	-
**2010–2011**	10 (51/2010-08/2011)	4347	1884	52.7%	47.3%	0.0%	10.0%	79.6%	10.5%	28.3%	1.8%	69.9%
**2011–2012**	5 (05/2012-09/2012)	3198	1279	96.2%	3.8%	0.0%	92.5%	4.4%	3.1%	30.6%	36.7%	32.7%
**2012–2013**	11 (51/2012-09/2013)	4025	2272	45.1%	54.9%	0.0%	41.4%	53.0%	5.6%	5.9%	69.7%	24.4%

^(a)^ These include all influenza cases, including those that were not subtyped/characterized.

One influenza virus type or subtype was clearly dominant (>60% of positive swabs) in eight out of ten seasons (Table 1): A(H3N2) in five seasons and seasonal A(H1N1), pandemic influenza A(H1N1)pdm09 and influenza B in one season each. Influenza virus co-circulation was observed in 2010–2011 (A(H1N1)pdm09 and B) and in 2012–2013 (A(H3N2), A(H1N1)pdm09 and B).

Influenza B represented more than 50% of reported influenza cases in 2005–2006 (62.5%) and 2012–2013 (54.9%). During the seven seasons when influenza B cases were reported, Victoria and Yamagata lineages predominated in three seasons each, while the two lineages co-circulated in 2011–2012.

**Fig 2:**
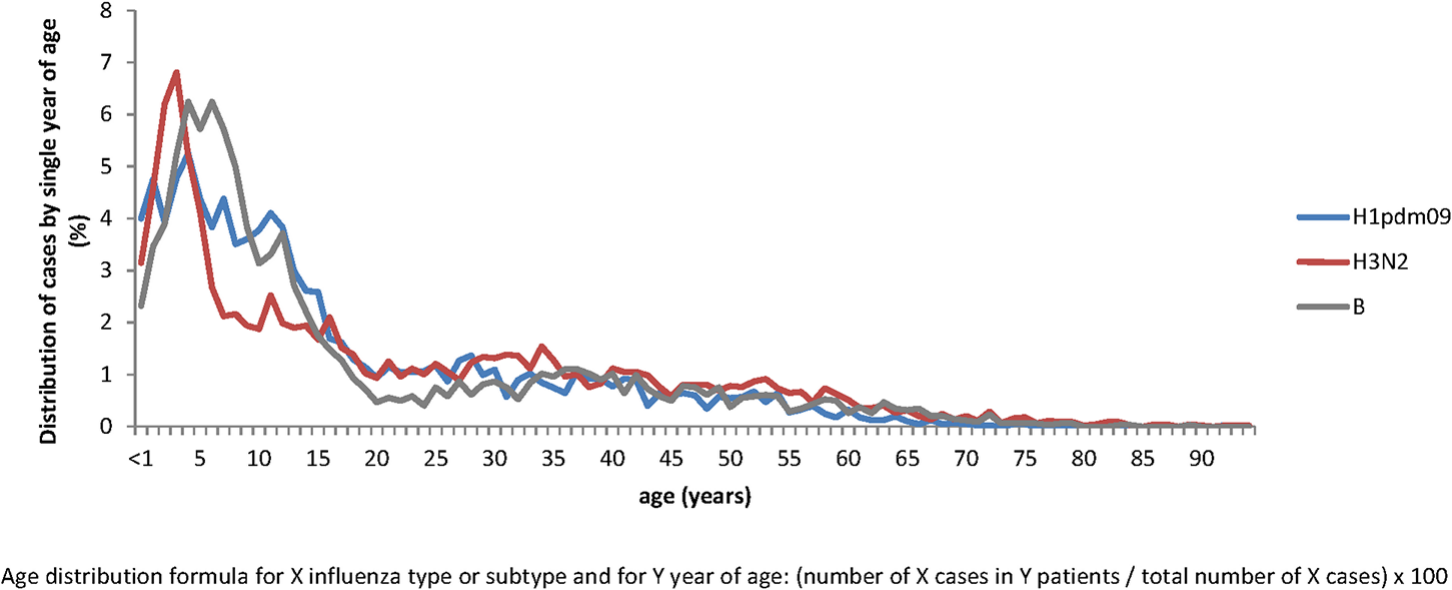
Age distribution of influenza cases by virus type and subtype. The IBVD (Influenza B in Vircases database) study, France, 2003–2004 to 2012–2013. The age distribution of H3N2 cases is shown in red, B cases in grey and H1pdm09 in blue.

The age distribution of patients infected with A(H3N2), A(H1N1)pdm09 and influenza B viruses is shown in Fig 2. The proportion of cases aged 0–14 or 15+ years was 49.2% and 50.8% for A(H3N2), 59.7% and 40.3% for A(H1N1)pdm09, and 62.7% and 37.3% for type B viruses. In particular, the Kolmogorov-Smirnov test revealed that the age distributions of patients infected with the different influenza viruses were significantly different (p<0.01). Both the mean and the median age of influenza A(H3N2) patients (21.6 and 15 years, respectively) were significantly different from B (18.2 and 10 years, p<0.01 for both comparisons) and A(H1N1)pdm09 (17.1 and 11 years, p<0.01 for both comparisons) influenza patients.

The median delay between onset of symptoms and consultation was 1 day among influenza A patients aged less than 65 years and influenza B patients aged 0–4 or 5–14 years, and 2 days for the other age groups irrespective of the virus type. The mean maximum temperature slightly decreased with age, with no clinically meaningful differences by type or subtype/lineage.

There were only few differences in the clinical presentation of influenza patients aged 0–4 years across different virus types and subtypes (Table 2). Compared to influenza B patients, influenza A patients had a slightly higher frequency of shivering and rhinitis, A(H3N2) patients a slightly higher frequency of rhinitis, while cough and expectoration were slightly more frequent among patients infected with the A(H1N1)pdm09 virus.

**Table 2:** Clinical presentation of influenza patients according to age group and influenza virus type and subtype in the age groups 0–4 years and 5–14 years. Univariate analysis. The IBVD (Influenza B in Vircases database) study, France, 2003–2004 to 2012–2013.

	0–4 years				5–14 years			
	B^(a)^	A^(a)^	A(H3N2)	A(H1N1)pdm09	B^(a)^	A^(a)^	A(H3N2)	A(H1N1)pdm09
	n = 728	n = 2711	n = 1163	n = 913	n = 1432	n = 3284	n = 1042	n = 1488
**Gender ratio (M/F)**	1.0	1.1	1.1	1.0	1.2	1.2	1.2	1.2
**% of patients with 7 symptoms or more**	22.5%	23.7%	22.5%	24.8%	47.0%	48.4%	51.1% *	45.8%
**Symptoms (%)** ^**(b)**^								
Fever	99.2%	99.0%	99.0%	99.1%	98.9%	98.6%	98.3%	98.7%
Asthenia	59.4%	60.2%	60.4%	63.8%	78.5%	77.7%	76.9%	80.2%
Myalgia	-	-	-	-	56.7%	57.4%	57.4%	57.9%
Shivering	33.8%	38.1% *	38.3%	36.4%	61.4%	63.6%	64.2%	61.5%
Cephalalgia	-	-	-	-	74.6%	74.1%	74.5%	74.4%
Cough	78.1%	79.9%	80.4%	83.5% **	82.1%	86.1% **	84.1%	89.6% **
Expectoration	9.6%	11.8%	9.5%	13.1% *	10.9%	13.4% *	11.9%	14.1% *
Bronchitis/bronchiolitis	6.0%	7.1%	7.1%	6.5%	5.6%	6.4%	6.2%	6.1%
Rhinitis	77.4%	80.9% *	83.4% **	75.4%	74.5%	72.2%	76.5%	65.7% **
Pharyngitis	49.9%	49.6%	50.6%	45.8%	55.0%	55.1%	60.9% **	48.6% **
Otitis/earache	14.4%	12.7%	12.5%	11.9%	5.6%	5.9%	7.7% *	4.3%
Gastrointestinal symptoms	20.0%	20.2%	19.7%	22.1%	24.9%	21.8% *	21.6%	21.3% *
Conjunctivitis	12.0%	13.1%	13.5%	10.0%	11.6%	11.6%	11.3%	9.8%
Adenopathy	10.7%	12.0%	11.0%	9.4%	14.7%	14.7%	15.5%	12.1%

^(a)^ These include all influenza cases, including those that were not subtyped/characterized.

^(b)^ sudden onset of symptoms is not presented as included in the ARI case definition; dyspnoea is not presented as only collected since the 2009–2010 season; myalgia and cephalalgia were not considered among children aged 0–4 years as they cannot report their subjective symptoms.

p-value for the comparison with influenza B patients of the same age group:

* <0.05

**<0.01

More statistically significant differences were observed in the age group 5–14 years (Table 2). Patients infected with an influenza A virus presented slightly more often with cough and expectoration, and less frequently with gastrointestinal symptoms. Patients infected with the A(H3N2) subtype had more frequently pharyngitis and otitis/earache, and presented with seven or more symptoms more frequently than patients infected with influenza B virus. The most notable differences were observed when comparing patients with A(H1N1)pdm09 and type B influenza virus infection: the latter had a higher frequency of rhinitis, pharyngitis and gastrointestinal symptoms, and were less frequently presenting with cough and expectoration.

Patients aged 15–64 years and infected with type A virus were slightly less frequently presenting with pharyngitis and otitis/earache than those infected with influenza B. Influenza A(H3N2) patients aged 15–64 years had a lower frequency of cough than influenza B patients of the same age (Table 3). Patients with A(H1N1)pdm09 virus infection aged 15–64 years were overall less symptomatic than patients with influenza B of the same age group, as they presented less frequently with cephalalgia, bronchitis/bronchiolitis, rhinitis, pharyngitis, otitis/earache and conjunctivitis.

**Table 3:** Clinical presentation of influenza patients according to age group and influenza virus type and subtype in the age groups 15–64 years and 65 years or older. Univariate analysis. The IBVD (Influenza B in Vircases database) study, France, 2003–2004 to 2012–2013.

	15–64 years				65+ years			
	B^(a)^	A^(a)^	A(H3N2)	A(H1N1)pdm09	B^(a)^	A^(a)^	A(H3N2)	A(H1N1)pdm09
	n = 1213	n = 4787	n = 2149	n = 1594	n = 73	n = 195	n = 129	n = 27
**Gender ratio (M/F)**	0.9	1	0.9	1	0.7	0.8	0.8	1.1
**% of patients with 7 symptoms or more**	68.4%	68.9%	71.5%	64.9% *	56.2%	53.9%	55.0%	55.6%
**Symptoms (%)** ^**(b)**^								
Fever	96.6%	96.3%	96.5%	96.2%	97.3%	95.8%	95.3%	92.6%
Asthenia	90.0%	88.4%	88.0%	88.9%	83.6%	80.4%	78.3%	85.2%
Myalgia	89.3%	89.1%	90.0%	87.0%	84.9%	77.3%	75.2%	85.2%
Shivering	82.4%	83.7%	84.7%	80.8%	77.5%	69.4%	69.5%	70.4%
Cephalalgia	81.5%	81.8%	83.1%	78.1% *	73.6%	65.5%	66.7%	55.6%
Cough	90.0%	88.4%	87.2% *	93.1% **	94.4%	89.2%	91.5%	88.9%
Expectoration	23.3%	24.7%	24.9%	23.1%	30.9%	31.7%	30.6%	42.3%
Bronchitis/bronchiolitis	8.9%	9.4%	10.5%	6.8% *	15.9%	16.7%	17.8%	15.4%
Rhinitis	76.4%	74.4%	77.7%	67.6% **	69.4%	76.3%	73.6%	74.1%
Pharyngitis	67.0%	61.0% **	62.6% *	55.1% **	46.5%	51.8%	48.8%	40.7%
Otitis/earache	9.3%	6.7% **	6.4% **	6.6% **	0.0%	3.1%	2.3%	3.7%
Gastrointestinal symptoms	15.8%	16.4%	17.1%	15.2%	17.1%	11.3%	10.1%	0.0% *
Conjunctivitis	11.5%	10.6%	11.8%	7.0% **	4.5%	6.9%	8.3%	3.7%
Adenopathy	9.0%	10.3%	11.5%	8.0%	6.9%	4.0%	2.1%	7.4%

^(a)^ These include all influenza cases, including those that were not subtyped/characterized.

^(b)^ sudden onset of symptoms is not presented as included in the ARI case definition; dyspnoea is not presented as only collected since the 2009–2010 season; myalgia and cephalalgia were not considered among children aged 0–4 years as they cannot report their subjective symptoms.

p-value for the comparison with influenza B patients of the same age group:

* <0.05

**<0.01

No significant differences in the clinical presentation of influenza A vs. B patients emerged among those aged 65 years or older, partly due to small numbers of influenza cases in this age group (Table 3).

Very few differences were observed in the clinical presentation of influenza B patients by lineage (Table 4). Most differences were observed for patients aged 5–14 years, among which those infected with a virus of the Victoria lineage had a higher frequency of cephalalgia, rhinitis and pharyngitis.

**Table 4:** Clinical Presentation of Influenza B Patients According to Age Group and Virus Lineage (Victoria versus Yamagata). The IBVD (Influenza B in Vircases database) Study, France, 2003–2004 to 2012–2013.

	0–4 years		5–14 years		15–64 years		>65 years	
	B Yamagata	B Victoria	B Yamagata	B Victoria	B Yamagata	B Victoria	B Yamagata	B Victoria
	(n = 318)	(n = 136)	(n = 415)	(n = 397)	(n = 488)	(n = 232)	(n = 36)	(n = 13)
**Gender ratio**	1.0	1.0	1.2	1.4	1.0	1.0	0.9	0.7
**% of patients with 7 symptoms or more**	21.4%	22.8%	41.2%	48.7% *	65.7%	64.2%	50.0%	53.9%
**Symptoms (%)** ^**(**^ ^**a**^ ^**)**^								
Fever	99.7%	100.0%	99.0%	98.5%	96.3%	97.0%	94.4%	100.0%
Asthenia	58.2%	64.7%	78.0%	77.6%	90.1%	88.8%	88.9%	69.2%
Myalgia	24.6%	28.7%	52.7%	57.9%	90.3%	87.5%	83.3%	92.3%
Shivering	31.8%	33.1%	59.6%	64.8%	83.2%	78.3%	80.6%	61.5%
Cephalalgia	26.2%	34.6%	71.7%	80.6% **	79.5%	81.5%	74.3%	84.6%
Cough	80.3%	82.4%	81.1%	84.9%	88.8%	90.1%	97.1%	92.3%
Expectoration	6.8%	12.5% *	10.0%	12.9%	23.3%	17.3%	32.4%	38.5%
Bronchitis/bronchiolitis	3.2%	4.4%	3.0%	4.3%	8.5%	6.1%	17.6%	23.1%
Rhinitis	77.5%	75.7%	65.9%	77.3% **	76.0%	75.8%	71.4%	61.5%
Pharyngitis	54.1%	52.9%	52.4%	61.5% **	65.0%	71.2%	37.1%	61.5%
Otitis/earache	14.7%	11.8%	6.5%	5.3%	9.9%	5.2% *	0.0%	0.0%
Gastrointestinal symptoms	19.9%	11.0% *	24.0%	21.4%	14.5%	17.2%	18.2%	15.4%
Conjunctivitis	10.6%	12.1%	8.1%	12.0%	11.1%	10.8%	0.0%	16.7% *
Adenopathy	10.6%	12.1%	15.1%	12.7%	7.0%	10.3%	4.0%	0.0%

^a^ Sudden onset of symptoms is not presented as included in the ARI case definition; dyspnoea is not presented as only collected since the 2009–2010 season.

p-value for the comparison with influenza B patients of the same age group:

* <0.05

**<0.01

In the multivariable logistic analysis, age was significantly associated with influenza type: compared to patients aged 0–4 years, those aged 5–14 years were more likely to have been infected with a type B virus (OR 2.15, 95% CI 1.87–2.47, p<0.001), while those aged 15–64 years were more likely to have been infected with a type A virus (OR 0.83, 95% CI 0.73–0.96, p = 0.010) (Table 5). Male gender was less frequently associated with influenza B (OR 0.89, 95% CI 0.80–0.98, p = 0.023). Influenza patients who were diagnosed during the influenza epidemic period were less likely to be infected by an influenza B virus (OR 0.38, 95% CI 0.33–0.43, p>0.001). The number of signs and symptoms recorded at presentation was not significantly associated with virus type.

**Table 5:** Variables associated with the odds of having been infected with influenza type B vs. A viruses. Multivariable logistic regression analysis adjusted by season. The IBVD (Influenza B in Vircases database) study, France, 2003–2004 to 2012–2013.

Characteristics	OR [95% CI]	*P* Value
Gender:		
Female	Reference	
Male	0.89 [0.80–0.98]	0.023
Age:		
0–4 years	Reference	
5–14 years	2.15 [1.87–2.47]	< .001
15–64 years	0.83 [0.73–0.96]	0.010
>65 years	1.12 [0.80–1.58]	0.501
No. of signs and symptoms ^a^:		
< 7	Reference	
≥ 7	0.94 [0.85–1.05]	0.287
Diagnosed during the epidemic period:		
No	Reference	
Yes	0.38 [0.33–0.43]	< .001

^a^ Signs and symptoms included were as follows: sudden onset of symptoms, asthenia, myalgia, shivering, cephalalgia, cough, expectoration, bronchitis/bronchiolitis, rhinitis, pharyngitis, otitis/earache, gastrointestinal symptoms, dyspnea, conjunctivitis, adenopathy.

## Discussion

The GROG is a network of general practitioners and paediatricians that routinely collects biological samples from patients presenting with ARI, allowing the IBVD study to rely on a very large number of influenza cases: 14,423 during ten consecutive epidemic seasons (2003–2004 to 2012–2013), of which 2,749 (19%) during the 2009–2010 pandemic season. These features of the GROG network, along with the good representativeness of practitioners participating in the surveillance scheme, ensure that the IBVD study could achieve its stated objective, which is to accurately describe the main characteristics and clinical presentation of medically-attended influenza patients according to virus type and subtype/lineage. Early characterization of circulating influenza strains in terms of clinical symptoms and complication rate is useful for clinicians in order to support the clinical decision and improve patients’ management.

The proportion of influenza cases due to different virus types and subtypes/lineages was highly variable during the period 2003–2004 to 2012–2013 in France. Whereas at least one influenza A virus subtype circulated during each season, influenza B viruses were almost absent (<1%) during three seasons (2003–2004, 2006–2007 and 2009–2010) and dominant (>50%) in two (2005–2006 and 2012–2013). During the 2003–2013 seasons, either the Victoria or the Yamagata lineage viruses were found to be the dominant B lineage, without any tendency for either one to prevail or wane in the long run, thus making it difficult to predict which lineage would be dominant during next season [23].

We found that influenza A viruses are more likely than influenza B viruses to be diagnosed during the epidemic period of influenza. This is not surprising, as influenza type A viruses account for most influenza cases during most seasons, and the epidemic season is usually defined as the period of the year when most influenza cases occur. However, whether influenza A and B differ in terms of timing of epidemics is a topic worth investigating, as it may have implications for vaccination programs.

As already mentioned, few studies were undertaken to investigate differences in the clinical presentation of influenza patients according to virus type and subtype/lineage that were truly population-based, sampling ARI (or influenza-like-illness, ILI) cases of all ages occurring in the community. In line with previously published reports [5–12], we found few differences in the clinical presentation of patients infected with type B vs. type A influenza virus, and these were in most cases inconsistent across age groups, thus confirming the statement by Kilbourne that “influenza is an unvarying disease caused by a varying virus” [24]. In particular, the frequency of a given symptom very rarely differed by 10% or more across patients infected with different virus types and subtypes. Only age may help discriminate between influenza A and B patients, as the latter are on average younger than the former. Although the frequency of reported symptoms at disease onset is similar across influenza virus types/subtypes and age groups, we have no information on the severity and evolution of signs and symptoms (nor on the frequency of more severe complications like pneumonia, hospitalization, and death), and we cannot rule out that differences might exist in this respect. Based on our findings, it is howeverunlikely that a bedside clinical prediction rule can be developed that allows a differential diagnosis, at onset of symptoms and with a good performance at population level, among patients affected by different influenza virus types.

Very few studies have compared the signs and symptoms of disease among influenza B virus infected patients by lineage [25]. The differences that we found were, once more, minimal and inconsistent across age groups. Despite the fact that the Victoria and Yamagata lineage viruses are genetically and antigenically distinct [26], the illness caused by viruses from either lineage is clinically indistinguishable.

Our study has strengths and limitations. The IBVD study relies on a representative nationwide network of sentinel physicians, with a high participation rate (73% for GPs, 76% for paediatricians) and characteristics comparable to those of other practitioners throughout France. Influenza patients who had been vaccinated or who had taken antivirals were excluded from the study because clinical presentation may be different and severity may be reduced among these patients [27]. As vaccinated individuals were not included in the dataset and persons over the age of 65 have a high vaccination rate in France, this age group may be underrepresented in our study. Likewise, institutionalized patients, patients that go directly to the emergency rooms of hospitals when ill, and people who less frequently consult a GP when suffering from mild disease [28] (like healthy French young adults, tourists, etc.) may be less likely to be included in the study database. However, there is little reason to suppose that the clinical presentation of influenza among these patients is different from study patients. Finally, children may be overrepresented in our study sample as they may be more likely than adults to consult and be sampled when presenting with ARI.

We have no information on prior exposure to related strains, which may also affect the clinical presentation. Patients consulting a practitioner because of ARI are not selected on the basis of frequency and/or severity of signs and symptoms, and the methodology of data collection has undergone only minor changes since the 2003–2004 season. Instead, the methods used for the virological diagnosis of influenza evolved during the study period. Whereas immunofluorescence, ELISA and culture were largely used before the 2009–2010 pandemic [29], RT-PCR has become the prime method in France thereafter [30]. As RT-PCR is the most sensitive technique [31], it is possible that the underestimation due to false negative results is higher before than after the pandemic. The number of differences that were statistically significant in Tables 2–4 was slightly higher than would be expected by chance alone, however the differences were small and not of clinical relevance, therefore there was no need to adjust for multiple comparisons.

As a consequence, the results reported here may not reflect the actual figures, if differences exist in the severity of illness caused by the different influenza virus types and subtypes/lineages. Moreover, differentiation of the clinical presentation may depend on the initial criteria to select patients for swabbing. Strictly speaking, our findings are representative of symptomatic cases of influenza that consult a GP and whose presentation at onset of symptoms fit the definition of ARI used in the IBVD study. This is an intrinsic limitation of all studies that rely on data collected within GP-based influenza surveillance systems, including ours.

In conclusion, the clinical illness produced by the different influenza virus types and subtypes shows no significant differences, in an outpatient setting, based on clinical presentation alone. However, different age groups may be preferentially affected by influenza during any given season depending on the pool of viruses that are circulating, which may result in a different disease burden.

## Supporting Information

S1 Fig Questionnaire for data collection within the IBVD (Influenza B in Vircases Database) study. (TIFF)
