# Supplementary figures and images for: Clinical Characteristics Are Similar across Type A and B Influenza Virus Infections
Source: PLoS One. 2015 Sep 1;10(9):e0136186. doi: 10.1371/journal.pone.0136186 (PMC4556513; doi:10.1371/journal.pone.0136186)

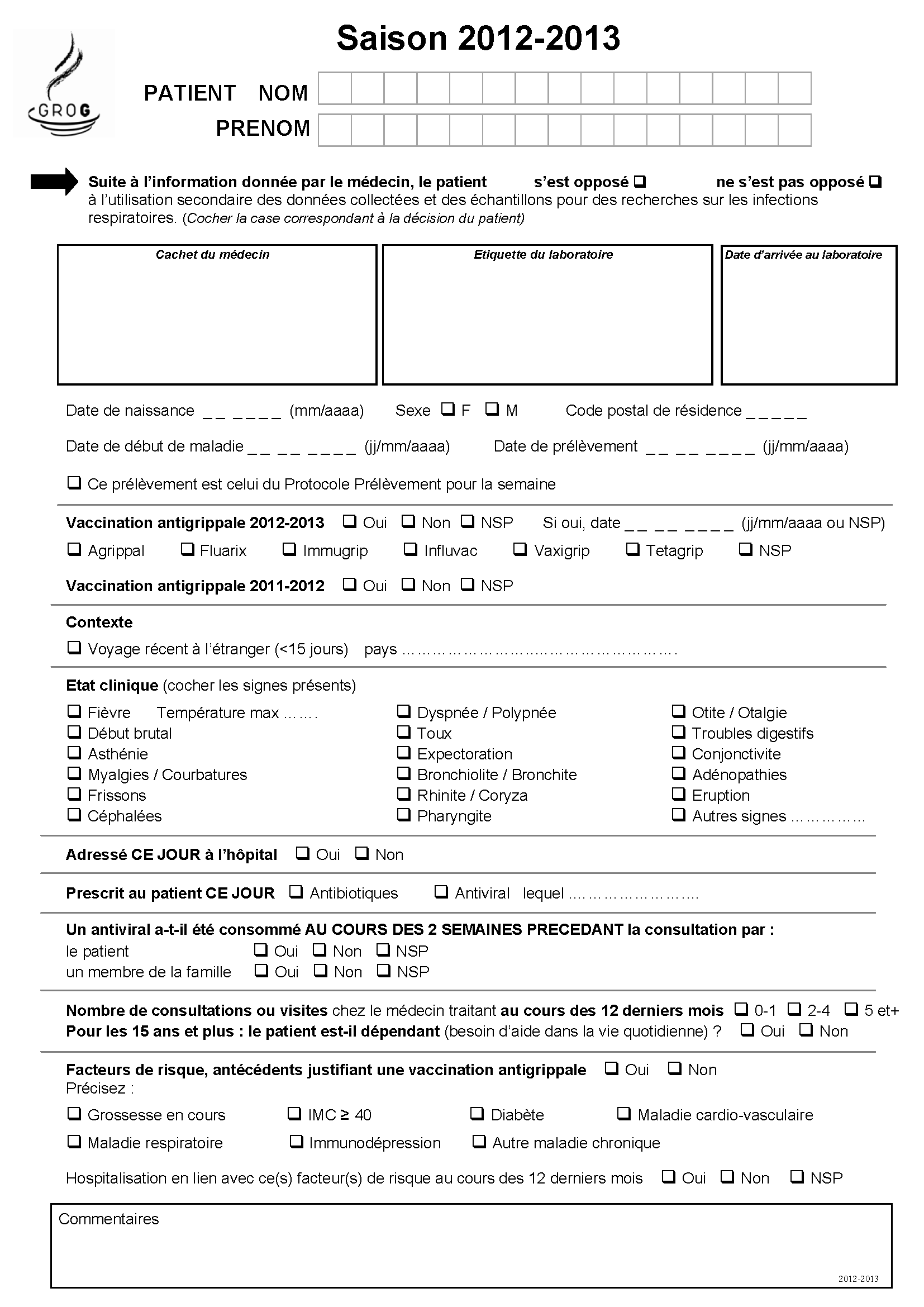

Supplement: S1 Fig — (TIFF) [file pone.0136186.s001.tiff]
